# Diagnostic Dilemma: Infective Endocarditis in the Setting of an Esophageal Abscess

**DOI:** 10.7759/cureus.47542

**Published:** 2023-10-23

**Authors:** Emad Elmusa, Ahmad Muneeb, Muhammad Waleed Raza, Hassan Tahir Khokhar, Nilmarie Guzman

**Affiliations:** 1 Internal Medicine, HCA Florida Orange Park Hospital, Orange Park, USA; 2 Infectious Disease, HCA Florida Orange Park Hospital, Orange Park, USA

**Keywords:** septic emboli, mssa bacteremia, transesophageal echocardiography (tee), esophageal abscess, infective endocarditis

## Abstract

Transesophageal echocardiography (TEE) is the preferred imaging modality to diagnose infective endocarditis (IE). However, esophageal disease can preclude performing a TEE. We present such a scenario. A patient with an esophageal abscess, methicillin-sensitive *Staphylococcus aureus *(MSSA) bacteremia, and septic pulmonary emboli with suspicion for IE based on the modified Duke criteria. However, due to the patient’s esophageal abscess, TEE could not be performed safely. We present this case to demonstrate a rare scenario in which a patient with an esophageal abscess also had presumed IE.

## Introduction

An intramural esophageal abscess is rare and typically develops secondary to esophageal mucosal injury [1]. Injury is usually traumatic, secondary to foreign bodies or instrumentation [2,3]. An esophageal abscess is not classically associated with infective endocarditis (IE) or bacteremia. We present a patient with an esophageal abscess, methicillin-sensitive *Staphylococcus aureus* (MSSA) bacteremia, pulmonary septic emboli, and suspicion for IE based on the modified Duke criteria. Transthoracic echocardiography (TTE) was performed and showed normal systolic function, no significant valvular disease, and no evidence of IE. However, due to the unreliability of TTE, inability to perform TEE because of patient safety, and clinical suspicion of IE, the patient was empirically treated for six weeks with intravenous (IV) cefazolin for presumed IE. After completing antibiotics, the patient reported resolution of symptoms.

## Case presentation

A 60-year-old female with a past medical history significant for essential hypertension, type 2 diabetes mellitus, and unspecified thyroid cysts status post right-sided thyroidectomy 23 years prior, presented with a chief complaint of progressive dysphagia to solids for the past two weeks. The patient also endorsed subjective fevers and shortness of breath. The patient returned from Ethiopia seven days ago, where she stayed for 24 days. The patient denied sick contacts. The patient does not remember ingestion of foreign bodies, including bones. The patient denied recent trauma, chest pain, or weight loss.

**Figure 1:**
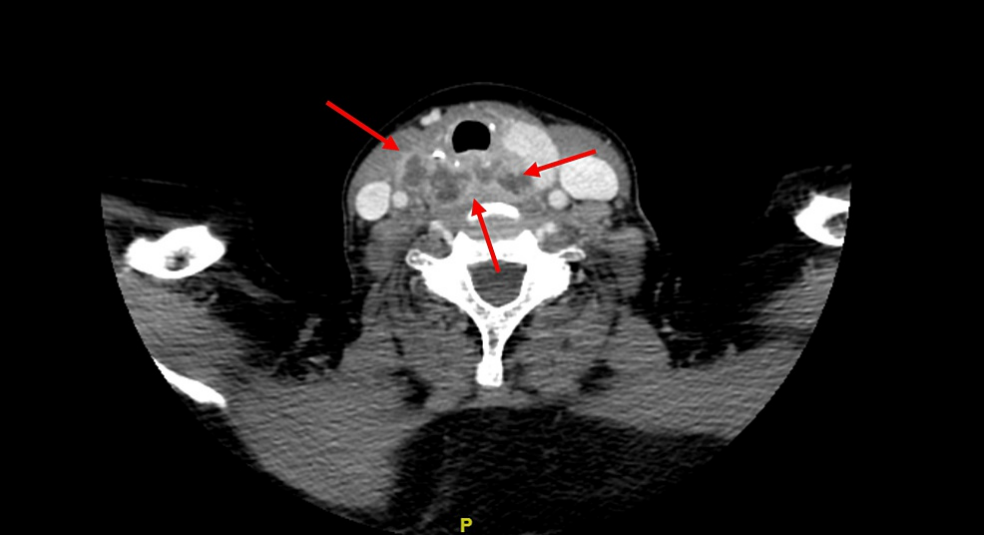
CT scan of the neck with IV contrast demonstrates a collection of hypodense rim-enhancing lesions interposed between the esophagus and trachea (red arrows).

**Figure 2:**
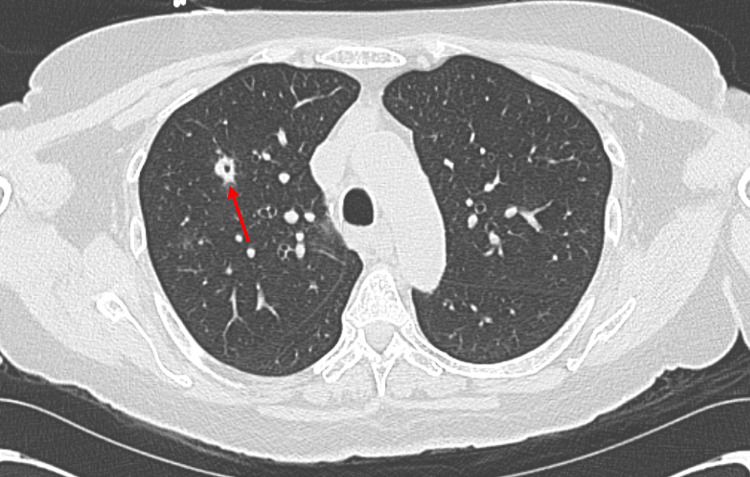
CT scan of the chest with IV contrast showcases a cavitary pulmonary nodule (red arrow).

On presentation in the emergency department, vital signs revealed a temperature of 98.7 °F, heart rate of 99 bpm, respiratory rate of 18 bpm, blood pressure of 147/80 mmHg, and oxygen saturation of 100% at room air. On physical examination, the patient’s oral, pulmonary, skin, and cardiac examinations were negative. Pertinent initial laboratory values are shown in Table 1. CT scan of the neck with IV contrast demonstrated a collection of hypodense rim-enhancing lesions interposed between the esophagus and trachea (Figure 1). CT scan of the chest with IV contrast showed peripheral and central pulmonary nodules - two of which had a cavitary component (Figure 2). Overall, findings were concerning for an esophageal abscess and septic pulmonary emboli.

**Table 1:** Laboratory values on initial presentation.

Laboratory test	Result	Reference range
White blood cell count (WBC)	11.0 x 10^3	Reference range: 4.0-10.5
Hemoglobin	12.8 g/dL	Reference range: 11.2-15.7 g/dL
Lactic acid	0.5 mmol/L	Reference range: 0.4-2.0 mmol/L
C-reactive protein	145.0 mg/L	Reference range: < 10 mg/L

The patient was treated empirically with vancomycin and ceftriaxone for broad-spectrum coverage. Two sets of blood cultures were drawn before antibiotic administration.

The gastroenterology service took the patient for an esophagogastroduodenoscopy (EGD). It showed an erythematous area with surrounding inflammation and purulent discharge in the upper third of the esophagus. No biopsy was taken due to concern for esophageal perforation.

At 24 hours, one out of two blood cultures grew MSSA. Based on the culture result, the patient’s antibiotics were changed to IV cefazolin 2 g every eight hours. The patient was presumed to have possible native valve IE based on the modified Duke criteria, meeting zero major but three minor clinical criteria (i.e., fever, vascular phenomena, and microbiological evidence).

The pulmonology service took the patient the following day for a bronchoscopy. Bronchoalveolar lavage (BAL) was performed and sent to the laboratory for culture. The BAL culture grew MSSA and was negative for *Mycobacterium tuberculosis*. Because of the esophageal abscess, the patient was unable to safely have a transesophageal echocardiogram. A transthoracic echocardiogram revealed normal systolic function, no significant valvular disease, and no evidence of IE.

The patient’s dysphagia slowly improved during her hospital stay. Repeat blood cultures 48 hours after initiation of IV antibiotics showcased no growth. Human immunodeficiency virus (HIV) 1 and 2 antibodies and HIV 1 p24 antigen were negative. The patient was discharged home on hospital day nine with a plan to continue IV cefazolin for a total of six weeks. The patient was instructed to follow up outpatient for TEE after completion of antibiotics.

The patient was followed for approximately six months after discharge by primary care. The patient reported that her symptoms completely resolved after completing six weeks of IV cefazolin. The patient did not follow up for TEE.

## Discussion

Infective endocarditis is an infection of the cardiac endothelium [4]. Interestingly, bacteremia commonly occurs after chewing or tooth brushing; however, the cardiac endothelium maintains its integrity [4]. Endothelial damage leads to the development of a platelet-fibrin-rich thrombus, which can act as a nidus for bacterial adherence [4]. Endothelial damage can occur in the setting of a prosthetic valve, rheumatic valve disease, or valve sclerosis [4]. Endothelial damage can also directly occur secondary to virulent bacteria, such as *S. aureus* [4].

The incidence of IE is reported to be approximately 10 per 100,000 [5]. IE complications include native or prosthetic valve obstruction or regurgitation, abscess formation, atrioventricular conduction block, congestive heart failure, cardiogenic shock, stroke, or metastatic infection [4,5]. *S. aureus *is the most common causative bacteria, with about 26.6% [6].

Diagnosis of IE can be challenging because it often necessitates diagnostic suspicion guided by the modified Duke criteria. Transthoracic echocardiogram yields a sensitivity of 75% and specificity of 90% in detecting vegetation [4,5]. The transesophageal echocardiogram yields a sensitivity and specificity exceeding 90% in detecting vegetation [4,5]. Therefore, with high clinical suspicion and an unrevealing TTE, TEE is necessary to rule out IE.

We present a patient with an esophageal abscess, MSSA bacteremia, and septic pulmonary emboli with suspicion for IE based on the modified Duke criteria. Initial TTE was negative for IE. However, because of the patient’s purulent esophageal abscess, it was unsafe to proceed with TEE. The patient was empirically treated for presumed IE in order to avoid complications of untreated IE. The patient’s symptoms completely resolved after completing six weeks of IV cefazolin. The patient did not follow up for outpatient TEE.

Of note, what connected an esophageal abscess and septic pulmonary emboli in the setting of MSSA bacteremia? This is a rare scenario. Bacterial infection of the esophagus is uncommon, and normal flora of the mouth or upper respiratory tract are usual culprits, including *S. aureus* [7]. In the absence of penetrating trauma, patients at risk of bacterial infection of the esophagus include immunocompromised patients [8-9]. Two clinical scenarios are likely: the patient had MSSA bacteremia and subsequently developed an esophageal abscess and IE with septic embolization to the lungs or the patient developed an esophageal abscess and then had bacteremia and subsequently developed IE with septic pulmonary emboli.

## Conclusions

Esophageal abscesses are rare and are uncommonly reported in the setting of infective endocarditis or bacteremia. The coexistence of these infections is problematic because performing a transesophageal echocardiogram in this setting can be a relative contraindication. However, due to clinical suspicion of IE and complications associated with untreated IE, we empirically treated our patient for six weeks with IV cefazolin. It is our goal to present this rare scenario in which a patient with an esophageal abscess also had presumed IE.
